# Supplementary material for: New bounded unit Weibull model: Applications with quantile regression
Source: PLoS One. 2025 Jun 10;20(6):e0323888. doi: 10.1371/journal.pone.0323888 (PMC12151439; doi:10.1371/journal.pone.0323888)
Supplement: Attachment — Submitted filename: Response to Reviewers.docx [file pone.0323888.s002.docx]

**New Bounded Unit Weibull Model: Applications with Quantile Regression**

Before we begin addressing the referee's comments point by point, we would like to thank him/her for his/her valuable feedback, which we believe has paved the way for a significant improvement of the paper. We will now present our responses to the specific comments.

• The referee comments are shown in black

• Our responses are shown in red color.

• All corrections of the revised version have been incorporated in red color.

Reviewers' comments:

Reviewer's Responses to Questions

**Comments to the Author**

**1. Is the manuscript technically sounds, and do the data support the conclusions?**

The manuscript must describe a technically sound piece of scientific research with data that supports the conclusions. Experiments must have been conducted rigorously, with appropriate controls, replication, and sample sizes. The conclusions must be drawn appropriately based on the data presented.

Reviewer #1: Yes

Reviewer #2: Yes

Reviewer #3: Yes

**2. Has the statistical analysis been performed appropriately and rigorously?**

Reviewer #1: Yes

Reviewer #2: Yes

Reviewer #3: Yes

**3. Have the authors made all data underlying the findings in their manuscript fully available?**

The [PLOS Data policy](http://track.editorialmanager.com/CL0/http:%2F%2Fwww.plosone.org%2Fstatic%2Fpolicies.action%23sharing/1/010f0195724d7703-f4d3ec70-bc54-4f6d-98be-e3479a4b71b1-000000/Ye6uP7Jo2GRqALUlFuk_wv6XeJHlmeTTNsLY2nlDMQU=201) requires authors to make all data underlying the findings described in their manuscript fully available without restriction, with rare exception (please refer to the Data Availability Statement in the manuscript PDF file). The data should be provided as part of the manuscript or its supporting information, or deposited to a public repository. For example, in addition to summary statistics, the data points behind means, medians and variance measures should be available. If there are restrictions on publicly sharing data—e.g. participant privacy or use of data from a third party—those must be specified.

Reviewer #1: Yes

Reviewer #2: Yes

Reviewer #3: Yes

**4. Is the manuscript presented in an intelligible fashion and written in Standard English?**

PLOS ONE does not copyedit accepted manuscripts, so the language in submitted articles must be clear, correct, and unambiguous. Any typographical or grammatical errors should be corrected at revision, so please note any specific errors here.

Reviewer #1: Yes

Reviewer #2: Yes

Reviewer #3: Yes

**5. Review Comments to the Author**

Please use the space provided to explain your answers to the questions above. You may also include additional comments for the author, including concerns about dual publication, research ethics, or publication ethics. (Please upload your review as an attachment if it exceeds 20,000 characters)

**Reviewer #1:**

Manuscript technically seems correct and the statistical analysis has been performed appropriately. It is presented in an intelligible fashion and written in Standard English. Quantile regression model is useful technique. It is also available on research square.

**Reviewer #2:**

This paper introduced a new bounded probability distribution derived from a transformation of the Weibull distribution. It examined key properties such as moments, entropy measures, and the quantile function. The authors developed the sequential probability ratio test (SPRT) and used the maximum likelihood estimation (MLE) method for parameter estimation. A Monte Carlo simulation assessed the performance of estimates. They also formulated a quantile regression model and applied it to real-world data on risk assessment and educational attainment. The results showed that the proposed model outperformed existing regression models in analyzing bounded variables.

However, the authors have considered a remarkable research work in a methodically structured manner. I have the following suggestions for improvement of the paper:

1. The authors should maintain uniformity in the use of abbreviations. If abbreviations are already defined, do not use the full form of them again.

Ans: Thank you for your valuable suggestion. We have revised the manuscript and corrected them.

2. Make the research gap section clear and highlight your work separately.

Ans: Thank you for your in-depth suggestion. We have presented the research gap in the introduction section in separate paragraph.

3. Please categorize your and previous research in the Table in the section Literature Review to show the better research gap.

Ans: Thank you for your valuable feedback. We have presented literature review, objective of the study and research gap in the introduction section in the separate paragraphs each.

**Reviewer #3:**

Review Report of the Abstract

Title of the Abstract: New Bounded Unit Weibull Model: Applications with Quantile Regression
Authors: Laxmi Prasad Sapkota, Nirajan Bam and Vijay Kumar

Summary of the Abstract: This paper introduces a new bounded probability distribution derived from a transformation of the Weibull distribution. It discusses key properties such as moments, entropies, and the quantile function. Additionally, the sequential probability ratio test (SPRT) was developed for the proposed model. The maximum likelihood estimation method was used for parameter estimation, with Monte Carlo simulations assessing estimation performance. A quantile regression model was formulated and applied to data sets in risk assessment and educational attainment, showing superior performance over alternative regression models. The findings contribute to the statistical toolkit for analyzing bounded variables across scientific fields.
**Strengths:**
1. Relevance: The abstract addresses an important topic within its field.
2. Clarity: The research problem, methodology, and conclusions are clearly presented.
3. Novelty: The study provides new insights by introducing a new bounded probability distribution.
4. Methodological Rigor: The use of SPRT, maximum likelihood estimation, and Monte Carlo simulation strengthens the research.

**Areas for Improvement:**

1. Objective Statement: The purpose of the study could be more explicitly stated.

Ans: Thank you for your valuable suggestion. The primary objective of this study is to propose a robust bounded probability model that can be effectively utilized across various fields, including sequential probability ratio testing (SPRT). Additionally, this study aims to develop a quantile regression model for analyzing real-world data, ensuring flexibility and applicability in diverse practical scenarios. We have incorporated this in introduction section.

2. Comparison with Existing Models: While the study mentions alternative models, more details on their limitations and the advantages of the new model would enhance clarity.

Ans: Thank you for your valuable feedback. We have now included a detailed comparison with existing models, highlighting their limitations and the advantages of our proposed model in introduction section and conclusion section.

Authors may consider Toppe-Leone Distribution, Unit Gamma, Unit logistic etc.

Ans: Thank you for your in-depth suggestion. Due to the length of the paper, we are unable to include these models this time. However, we will consider incorporating them in future studies.

Mazucheli, J., Menezes, A. F. B., & Dey, S. (2018). Improved maximum-likelihood estimators for the parameters of the unit-gamma distribution. Communications in Statistics-Theory and Methods, 47(15), 3767-3778.

Menezes, A. F. B., Mazucheli, J., & Dey, S. (2018). The unit-logistic distribution: Different methods of estimation. Pesquisa Operacional, 38(3), 555-578.

Shekhawat, K., Sharma, V.K. An Extension of J-Shaped Distribution with Application to Tissue Damage Proportions in Blood. Sankhya B 83, 548–574 (2021).

Mazucheli, J., Menezes, A. F. B., & Chakraborty, S. (2019). On the one parameter unit-Lindley distribution and its associated regression model for proportion data. Journal of Applied Statistics, 46(4), 700-714.

Recommendation:
• Accept.
